# Lipid Oxidation and Colour Stability of Lamb and Yearling Meat (*Muscle longissimus lumborum*) from Sheep Supplemented with Camelina-Based Diets after Short-, Medium-, and Long-Term Storage

**DOI:** 10.3390/antiox10020166

**Published:** 2021-01-22

**Authors:** Eric N. Ponnampalam, Kym L. Butler, Stephanie K. Muir, Tim E. Plozza, Matthew G. Kerr, Wayne G. Brown, Joe L. Jacobs, Matthew I. Knight

**Affiliations:** 1 Animal Production Sciences, Agriculture Victoria Research, Department of Jobs, Precincts and Regions, AgriBio, Bundoora, VIC 3083, Australia; Matthew.Kerr@agriculture.vic.gov.au (M.G.K.); Wayne.Brown@agriculture.vic.gov.au (W.G.B.); 2 Biometrics Team, Agriculture Victoria Research, Department of Jobs, Precincts and Regions, Hamilton, VIC 3300, Australia; Kym.Butler@agriculture.vic.gov.au; 3 Animal Production Sciences, Agriculture Victoria Research, Department of Jobs, Precincts and Regions, Hamilton, VIC 3300, Australia; stephanie.muir@agriculture.vic.gov.au (S.K.M.); Matthew.Knight@agriculture.vic.gov.au (M.I.K.); 4 Plant Production Sciences, Agriculture Victoria Research, Department of Jobs, Precincts and Regions, Macleod, VIC 3085, Australia; Tim.Plozza@agriculture.vic.gov.au; 5 Animal Production Sciences, Agriculture Victoria Research, Department of Jobs, Precincts and Regions, Ellinbank, VIC 3821, Australia; Joe.Jacobs@agriculture.vic.gov.au; 6 Centre for Agricultural Innovation, School of Agriculture and Food, Faculty of Veterinary and Agricultural Sciences, The University of Melbourne, Parkville, VIC 3010, Australia

**Keywords:** feeding systems, diets, sheep, meat preservation, meat quality, antioxidant action, muscle, vitamin E

## Abstract

This study investigated the impact of feeding pelleted diets containing camelina (*Camelina sativa* L. Crantz) hay (CAHP) or camelina meal (CAMP) as a supplement compared with a control pellet (CONP) diet, without vitamin E fortification. The fatty acid profile, retail colour, and lipid oxidative stability of lamb and yearling meat (*m. longissimus lumborum*) stored for short-, medium-, or long-periods (2 days (fresh), 45 days and 90 days) under chilled to semi-frozen conditions were determined. The CAMP diet altered key fatty acids (*p* < 0.05) in a nutritionally beneficial manner for human health compared to the other diets, with increased total omega-3, decreased omega-6 fatty acids and decreased omega-6/omega-3 ratio of muscle. Muscle vitamin E concentration was lower (*p* < 0.05) for both camelina diets (CAMP and CAHP) when compared with the CONP diet, with the average concentrations less than 1 mg/kg muscle for all three treatments. Animal type and storage length were factors that all affected (*p* < 0.05) colour and lipid oxidative stability of meat. These results emphasise the importance of vitamin E concentration in meat stored for extended periods under semi-frozen conditions to maintain desirable meat colour during retail display, and to avoid off-flavour development of the cooked meat.

## 1. Introduction

Emerging economies in Asia and the Middle East have seen a dramatic increase in the amount of Australian sheep meat exported to these markets in recent years. In 2018/2019, Australia exported 447,000 tonnes of sheep meat to international markets as either frozen or refrigerated products [1]. For the Australian sheep meat industry to continue to grow exports, and meet consumer and safety regulator expectations, it is worth reviewing current production and processing techniques. Red meat preservation techniques commonly involve vacuum packing of primal cuts under anoxic conditions and then transporting the products at an appropriate temperature [2,3]. Longer storage periods and different storage conditions may be required for products to reach different export destinations with desired quality. For Asian and Middle Eastern markets, it might be most appropriate to transport sheep meat products under chilled (2 °C to 4 °C) or semi frozen (−0.5 °C to 0 °C) conditions. Maintaining sheep meat products under these conditions might be a better balance of limiting microbial spoilage and limiting energy consumption during transport when compared to either refrigerated, or frozen, meat products [2].

Red meat is a rich source of key nutrients such as protein, trace elements, vitamins and essential fatty acids (FAs) [4]. A critical component of managing the preservation or storage of red meat is an understanding of factors that affect the nutritional value and sensorial quality of the meat [5,6,7]. This is because considerable changes in the structural, functional and biochemical properties of red meat can occur during storage, and this can have a substantial effect on resultant product quality and integrity [8]. Factors associated with the production system, such as diet and animal types, can affect carcass fatness, nutritional value of meat, and meat sensory characteristics [5] by influencing structural and biochemical components of muscle tissues.

In summer and early autumn, Australian livestock producers rely on the grazing of senesced pastures, cereal hays, or stubble. These pasture or forage based grazing systems are often supplemented with available cereal grains or pulses to maintain animal productivity and performance. Camelina (*Camelina sativa* L. Crantz) forage hay and/or meal may provide an alternate option for improving ruminant animal production and nutritional value of meat (essential fatty acid) in seasons of low pasture availability. Previous studies have examined the growth and reproductive performance of replacement beef heifers fed camelina biodiesel co-products [9] and the fatty acid composition of blood in milking sheep supplemented with camelina cake [10]. Others showed that supplementing lambs with camelina cake or meal [11,12] and either chemically treated camelina seed or camelina oil [13] improved nutritionally desired unsaturated fatty acid content in the *longissimus* muscle. However, the effect of feeding camelina hay or meal as an unprotected (natural feed) supplement in a sheep finishing diet on the functionality (lipid oxidation and colour stability) of stored meat is unknown.

The aim of the study was to examine impacts of supplementing a finishing diet, which has nutritional characteristics typical of Australian production systems, with camelina hay or camelina meal without vitamin E fortification. Impacts examined included muscle vitamin E concentration, fatty acid profile, and flow on effects for retail colour stability and lipid oxidation of meat (*M. longissimus lumborum*, LL) stored for short-, medium-, or long-term under chilled to semi-frozen conditions. Maternal Composite (Composite) lambs and Merino yearlings were used in order to examine the effects on lamb and yearling meat, respectively.

## 2. Materials and Methods

### 2.1. Diets, Animals, and Experimental Design

Three pelleted diets were formulated (J.T. Johnson & Sons and TMR Feed Solutions, South Australia) using ingredients available in the major sheep producing regions of southeast Australia. Pelleted diets containing camelina hay (CAHP) and camelina meal (CAMP) were compared with a control diet containing cereal hay and grains (CONP). Feeds offered were recorded daily and refusals measured weekly. Feed samples were collected weekly over the course of the experiment and the samples bulked by treatment. These samples were dried at 100 °C, ground using a UDY Cyclone Mill (model # 3010-019) equipped with 1 mm sample screen and then analysed for chemical composition determined by near infra-red spectrometry (NIR, ACE Laboratories Pty. Ltd., Bendigo, Victoria, Australia). NIR prediction equations developed by Cumberland Valley Analytical Services (www.foragelab.com) using in house chemistry and NIR spectra using WinISI (Foss) chemometric software were used to determine the chemical constituents as reported in detail [14]. The ingredients used to formulate the experimental diets and the nutritive composition of each diet, including vitamin E, α-linolenic acid (n − 3) and linoleic acid (n − 6) concentrations, are presented in Table 1. Dried, ground, and homogenised feed samples were used to determine the vitamin E (1 g feed sample), α-linolenic acid, and linoleic acid (0.5 g feed sample) concentrations, with the analytical procedures used being the same as for muscle samples reported in Section 2.3. The metabolisable energy (ME) and crude protein (CP) concentrations of diets were about 11 MJ/kg dry matter (DM) and 15% crude protein (CP), respectively. Diets were designed to achieve at least 150 g/day of live weight gain (LWG) for both Composite lambs and Merino yearlings.

The study was approved by the Animal Ethics Committee of the Department of Jobs, Precincts and Regions (AEC Approval No: 2016-17). All procedures were conducted in accordance with the Australian Code of Practice for the Care and Use of Animals for Scientific Purposes [15]. Eighty Composite lambs (28–38 kg) and 80 Merino yearlings (37–43 kg) were selected from the Research flock maintained at Agriculture Victoria, Hamilton, VIC, Australia. At the commencement of the experiment, Composite lambs and Merino yearlings were 4 and 15 months of age, respectively. The study utilised 20 pens in the animal house facility at Agriculture Victoria, Hamilton. Each pen contained two feed troughs (21 cm width × 84 cm length × 51 cm height), with each trough capable of holding approximately 10 kg DM. For each animal type, three pens were used for the CAHP diet, three pens were used for the CAMP diet, and four pens were used for the CONP diet. Within each sheep type, animals were allocated to pens (eight lambs per pen) by stratified randomisation based on live weight. Animals always had free access to water.

### 2.2. Slaughter of Animals and Muscle Sample Collection

Animals were slaughtered at a commercial abattoir after ~3 h of transportation and 18 h in lairage, in similar numbers and allocation strata from each pen, after either 8, 9 or 10 weeks feeding as they reached an average slaughter weight of 51.4 kg for Merino yearlings and 52.5 kg for Composite lambs. At 24 h post-slaughter, carcasses were split in half and the left side of the carcasses were dissected between the seventh/eighth rib to the caudal end of the LL muscle. Muscle samples (LL) were collected for the measurement of vitamin E concentration (10 g, ~1 cm length), fatty acid profile (40 g, ~2 cm length), meat tenderness (60–65 g, ~3–4 cm length), and colour stability of short- (2 days, 80 g), medium- (45 days, 80 g), or long- (90 days, 80 g) term storage, respectively. The samples taken for colour stability were ~4 cm length. For animals slaughtered at 10 weeks (60 animals in total), two LL muscle samples (20 g) were collected to assess the lipid oxidative stability of short-, medium- and long-term storage of meat. All muscle samples were vacuum packed upon collection. Short-term storage (2 days) muscle samples used for colour evaluation were stored at between 2 and 3 °C. The 45-day and 90-day muscles samples were stored at between −0.5 °C to 0 °C until simulated retail display. The samples collected for vitamin E concentration, meat tenderness, fatty acid concentration, and lipid oxidation determination were stored at −20 °C until analyses were undertaken.

### 2.3. Determination of Meat (Muscle LL) Tenderness, Vitamin E Concentration, and Fatty Acid Composition

Meat tenderness was measured using a Warner–Bratzler shear force (WBSF) instrument according to Hopkins and others [16]. Extracted LL muscle (60–65 g) was vacuum packed and aged for 5 days at 2–3 °C. After 5-days ageing, the vacuum-packed sample was stored at −20 °C until meat tenderness analysis. Directly from frozen, the vacuum-packed sample was cooked at 70 °C for 35 min and immediately cooled in ice-slurry for 20 min. The sample was then removed from the vacuum-pack, padded dry and placed onto a tray. The tray was covered with film/wrap and refrigerated overnight at 3–4 °C. The next morning, meat tenderness measurements were performed using a Lloyd texture instrument (Model LRX, Lloyd Instruments, Hampshire, UK) fitted with a Warner-Bratzler shear blade moving at 300 mm/min crosshead speed. The thickness of the V shaped blade cutting edge is 0.88 mm. For each sample, three cuboidal strips with a cross section of 1 cm^2^ and 3 cm long were removed perpendicular to the muscle fibre direction. The average peak force value for each sample was measured from the WB shear instrument with values recorded in Newton. The data from the three samples were averaged to give a final shear force value for each animal. Muscle fatty acid concentration was determined using the procedure described by O’Fallon et al. [17], with modifications. Specifically, 20 g frozen meat samples were freeze dried, ground and a representative sub-sample (0.5 g) was processed for fatty acid extraction, methylation and gas chromatography (GC) quantification using a Varian 3800 GC instrument fitted with a FID detector (Agilent Pty. Ltd., Mulgrave, VIC., Australia). Fatty acid concentrations of meat were reported in mg/100 g meat (Table 2) as per nutrient reference for human dietary recommendations [4]. The vitamin E (α-tocopherol) concentration of meat was determined (1.00 ± 0.05 g freeze dried sample) using the method of Mestre Prates, Gonçalves Quaresma [18] and the results expressed as mg/kg of fresh meat.

### 2.4. Assessment of Colour and Lipid Oxidative Stability of Meat

After 2- (fresh), 45-, and 90-day storage, the designated 80 g portion of LL muscle was sliced into two slices of meat per sample, each slice ~2 cm in thickness, to expose a fresh meat surface. Both slices of meat were placed on a black foam tray and over wrapped with a PVC food film (15 μm thickness) as retail display packs. Trays were maintained at 3 °C–4 °C under fluorescent light (1000 lux) for 72 h to simulate retail display conditions. After blooming for 60 min, the redness (a*-value) of meat was measured in duplicate on each sample at 1 h, 24 h, 48 h, and 72 h using a Hunter Laboratory Mini Scan XE Plus meter with a 25 mm aperture, light source set to illuminant D-65 with a 10-degree standard observer (Model 45/0-S, Hunter Associates Laboratory Inc., Virginia, USA) [19]. The lipid oxidation of meat was obtained in malondialdehyde equivalents (mg MDA/kg of meat) using a thiobarbituric acid reactive substances (TBARS) procedure [20]. The lipid oxidation of meat was assessed at 1 h and 72 h of retail display time for meat stored for 2, 45, or 90 days under chilled to semi-frozen conditions.

### 2.5. Statistical Analyses

Except for one animal that died prior to slaughter due to unrelated causes, nearly all animals contributed to the statistical analyses of all measurements, apart from TBARS. Approximately 6% of the TBARS measures at 1 h, and 6% of the meat tenderness measurements could not be performed due to insufficient sample availability. A single 45-day storage TBARS at 72 h display reading and a single vitamin E reading were also not available. For each measurement determined on meat samples collected in this study, except TBARS, the values were first averaged over all animals from a pen that were slaughtered on the same day (two or three animals per pen at weeks 8 and 9; a total of five animals per pen in weeks 8 and 9 combined; three animals per pen at week 10). A single value for a pen was then obtained by averaging over the three kill day values for that pen. For TBARS measurements, the pen value was the average of the three carcasses obtained at the week 10 slaughter. These pen values were the unit of analysis for all measurements.

Vitamin E concentration, meat tenderness, and fatty acid measurements were analysed as a fully randomised two animal type × three diet factorial analysis of variance, which had 14 residual degrees of freedom. Redness of meat was analysed as a split plot repeated measurement analysis of variance with Greenhouse-Geisser correction for degrees of freedom, so that effects involving storage type and display time could be evaluated (Table 3). TBARS measurements were analysed similarly to redness, except that there were two display times, and thus no Greenhouse-Geisser correction was necessary (Table 4). Statistical analysis was carried out using the ANOVA directive and AREPMEASURES procedure in GenStat, version 19 [21].

## 3. Results

With the exceptions of a few FAs with 0.01 < *p* < 0.05 (Table 2), and a three way diet × animal type × display time interaction for a*-value (*p* = 0.004; Table 3), there was no evidence (*p* < 0.05) of any interaction between animal type and diet for any of the measurements (Table 2, Table 3 and Table 4). Thus, the results of diet and animal type, including interactions with storage type and display time, are reported separately.

### 3.1. Meat Tenderness, Vitamin E Concentration, and Fatty Acid Composition of Meat

In this study, no difference in meat tenderness (Warner–Bratzler shear force) was observed among animals fed the CONP, CAHP, or CAMP diet (*p* = 0.47; Table 2) or between the Composite lambs and Merino yearlings (*p* = 0.24; Table 2). The mean values for tenderness of meat was 35 N. Dietary vitamin E concentration in CAHP and CAMP diets was lower than the CONP diet (Table 1). This resulted in lower vitamin E concentration of meat in the CAHP and CAMP diets than the CONP diet (0.81, 0.86, and 0.99 mg/kg meat, respectively; *p* < 0.001; Table 2).

Compared to the CONP diet, the CAMP diet affected polyunsaturated fatty acid concentration, mainly via alpha-linolenic acid (ALA, 18:3n−3) and arachidonic acid (AA, 20:4n−6) concentrations in meat (Table 2). Alpha-linolenic acid concentration in meat of CAMP was approximately double the ALA concentration in meat of CONP (55 vs. 25 mg/kg meat; Table 2), presumably because the dietary ALA concentrations for CONP and CAMP were 228 and 846 mg/100 g of diet (Table 1). Arachidonic acid concentration in meat of CAMP was approximately 20% less than the AA concentration in meat of CONP (28 vs. 35 mg/kg meat, Table 2). There was no evidence of any difference between the CAHP diet and CONP diet for ALA concentration, total omega-3 concentration, total omega-6 concentration or the ratio of n−6/n−3 in meat, although AA was about 10% less in CAHP than CONP. Diet also affected long chain omega-3 FAs of eicosapentaenoic acid (EPA, 20:5n−3), docosapentaenoic acid (DPA, 22:5n−3) or docosahexaenoic acid (DHA, 22:6n−3) but the magnitude of these effects is smaller. The total muscle fat content was 65% (95% confidence interval = (57%, 73%)) greater for Merino yearlings than Composite lambs (4.88 vs. 2.97 g/100 g of meat). This resulted in higher values for most individual and major fatty acid groups in Merino yearlings when compared to Composite lambs (Table 2).

### 3.2. Stability of Meat Colour and Lipid Oxidation after 2-, 45-, and 90-Day Storage under Chilled to Semi-Frozen Conditions

**Figure 1:**
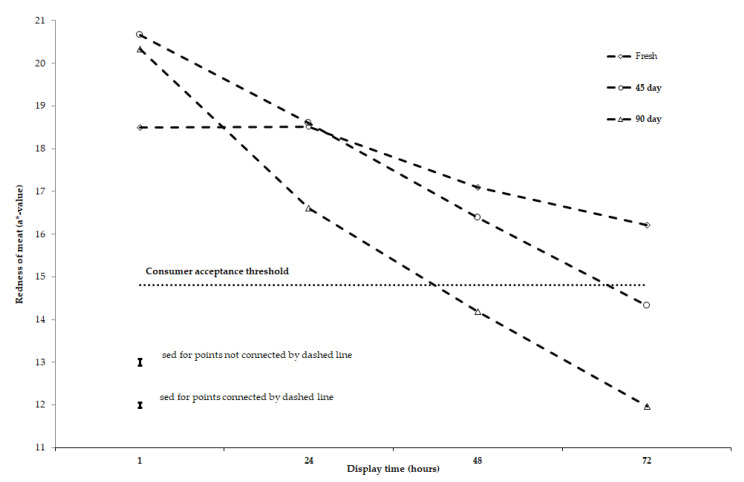
Effect of short-, medium-, and long-term storage (fresh, 45- and 90-day storage) on redness (a*-value) of muscle *longissimus lumborum* during 72 h of retail display under simulated refrigeration. Display time is the number of hours after muscle is cut and displayed for retail colour measurement. Sed denotes standard of difference.

**Figure 2:**
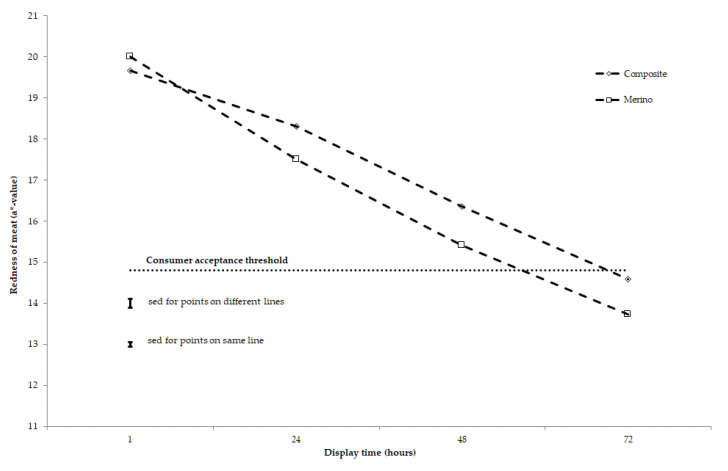
Effect of animal type (Composites vs. Merinos) on redness (a*-value) of muscle *longissimus lumborum* during 72 h of retail display under simulated refrigeration. Display time is the number of hours after muscle is cut and displayed for retail colour measurement. Sed denotes standard error of difference.

Redness of meat was affected by animal type, storage length, and display time, but not by diet in this study (Table 3). The redness of meat stored for a short duration (2 days, i.e., fresh meat) was above the threshold value of 14.8 for consumer acceptance after 72 h simulated retail display [22]. Meat stored for 45 days (medium-term) under semi-frozen conditions failed to maintain its redness past 48 h retail display and meat stored for 90 days (long-term) under semi-frozen conditions failed to maintain its redness past 24 h retail display (Figure 1). With animal type, the decline in meat redness with increasing display time was greater in yearling meat from Merino sheep than lamb meat from Composite sheep (*p* < 0.0001 for animal type × display time interaction; Table 3). Most of this greater decline in yearling meat from Merino sheep occurred between 1 and 24 h retail display (Figure 2).

**Figure 3:**
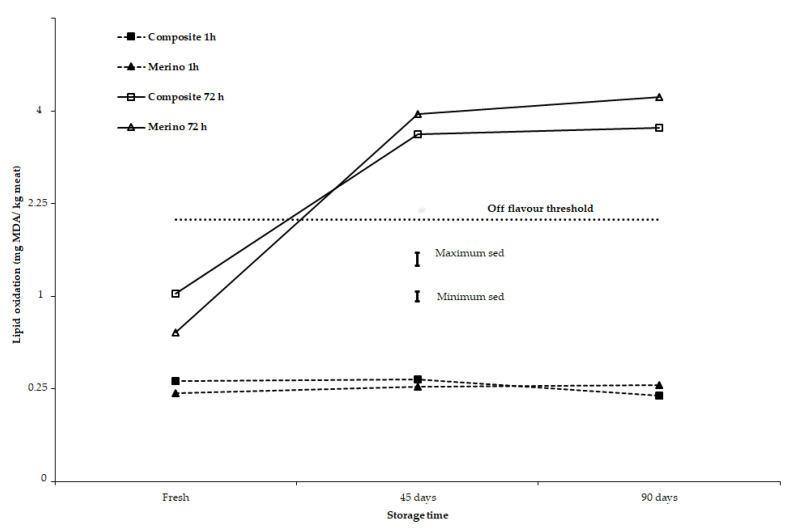
Effect of storage time (fresh, 45 and 90 days), retail colour display time (1 and 72 h) and animal type (lamb meat from Composite sheep and yearling meat from Merino sheep) on lipid oxidation (TBARS, mg MDA/kg meat). Results are presented on a square root scale along with the minimum and maximum standard errors of difference (sed) on that scale.

**Figure 4:**
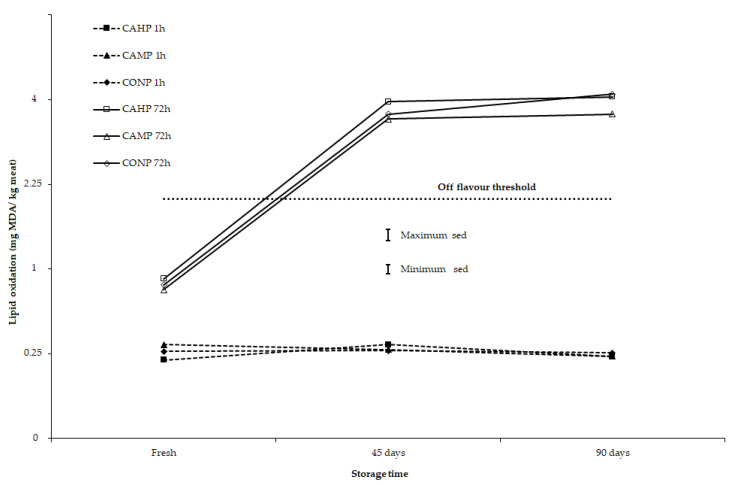
Effect of storage time (fresh, 45 and 90 days), retail colour display time (1 and 72 h) and diet (CAHP = pelleted diet containing camelina hay, CAMP = pelleted diet containing camelina meal and CONP = control diet containing cereal hay and grains) on lipid oxidation (TBARS, mg MDA/kg meat). Results are presented on a square root scale along with the minimum and maximum standard errors of difference (sed) on that scale.

It should be noted that for all storage lengths applied in this study, including lamb and yearling meat samples stored under chilled (2 days) and semi-frozen conditions (45 and 90 days), the lipid oxidation of meat at 1 h display time as assessed by TBARS was below the threshold that can develop abnormal flavour or other sensory defects in sheep meat. In terms of animal type (Composites vs. Merinos), there is evidence that at 72 h display time, lamb meat from Composite sheep had somewhat greater lipid oxidation than yearling meat from Merino sheep for meat stored for 2 days. However, for yearling meat from Merino sheep stored for 45 or 90 days, these samples had somewhat greater lipid oxidation than lamb meat from Composite sheep (*p* = 0.046 for 3-way interaction of animal type × storage time × display time; Table 4, Figure 3). At 72 h retail display, TBARS values for 45-day and 90-day storage meat samples were 10 to 15 times greater than the 1 h TBARS values (Figure 3 and Figure 4).

## 4. Discussion

With increasing international demand for premium red meat products, the storage conditions under which red meat products are stored and transported are becoming increasingly important. New production and processing techniques are required to extend the shelf life of premium products under chilled to semi-frozen conditions (for example, 3 months for sheep meat and up to 6 months for beef) between slaughter and consumption [2,3,23]. The maintenance of the safety and sensory attributes of meat during these extended storage periods is important [7]. Animal nutrition (diet) and animal type (breed) can influence carcass fatness and muscle (macro and micro) nutrients, which can, in turn, affect the colour and lipid oxidative status of the finished meat product when kept for different storage times and display temperatures [5,24]. These factors ultimately affect the profitability of the red meat industry as consumer perception, at the time of purchase of meat, can be influenced by the colour and sensory attributes of meat from previous eating experience. Therefore, it is important to determine the retail colour stability and lipid oxidative stability of meat after longer storage conditions.

To meet consumer acceptability for Australian lamb, meat tenderness below approximately 27 N is required [25]. Others [26] reported that meat tenderness above this threshold had a negative relationship with all consumer sensory scores (i.e., overall liking, juiciness, tenderness, flavour, odour, and taste of lamb). The average tenderness of lamb (33.3 N) and yearling (35.9 N) meat from all diets was consistent with previous studies where lambs were fed diets with oaten hay and cereal grain or oaten hay and protein supplements [27], and slightly higher than the 27 N required to meet consumer acceptability [25]. It is interesting to note that meat tenderness was not appreciably affected by animal type even though the Merino yearlings had remarkably higher muscle fatness compared with Composite lambs. One would have expected yearling meat from Merino sheep to be tenderer than lamb meat from Composite sheep due to this difference in total fat content (increased intramuscular fat content). This indicates that intramuscular fat content is not the only contributor to meat tenderness and other factors such as myofibrillar cross linkages, collagen content, and protease enzymes [28,29] might be involved with the relationship between meat tenderness and age of animals. As occurred in a previous study that examined the inclusion of camelina meal in a concentrate diet [30], inclusion of camelina did not affect meat tenderness of finished lambs.

The average vitamin E concentration differed between Composite sheep (0.8 mg/kg lamb meat) and Merino sheep (1.0 mg/kg yearling meat). Whilst these values are low, they are typical of previous studies that have finished sheep on traditional Australian feedlot diets [5,31]. In turn, vitamin E concentration of meat obtained from sheep fed the concentrate diets without vitamin E supplementation are generally between 0.5 and 2 mg/kg [32,33,34,35]. In contrast, in Australia, 4 month old weaned lambs finished on green pasture for 6 weeks had a vitamin E concentration of 2.1–2.9 mg/kg meat [5]. The optimum level for vitamin E in meat to delay lipid oxidation, and thus maintain retail colour, of sheep meat varies between studies. Previous studies have shown that the colour of meat was not affected when vitamin E concentration of meat is between 3.2–3.6 mg/kg meat [36] or between 3.5 and 4.0 mg/kg [37]. Moreover, lipid oxidation was reduced in sheep meat when the vitamin E concentration of meat was greater than 3.45 mg/kg [38]. Yearling meat from Merinos had a slightly higher vitamin E concentration than lamb meat from Composite sheep. One potential explanation for this result is that the Merino yearlings were exposed to green spring pasture twice in their lifetimes, whereas Composite lambs had been exposed to green spring pasture only once. This extra growing period for Merino yearlings is normal practice because Merino sheep grow slower and achieve market weight at older age than faster growing crossbred lambs [39,40]. Thus, over their lifetime, the Merino yearlings had an opportunity to consume more green pastures that are rich in vitamins and essential FAs, which also emphasises that the feeding length of ruminants on green pasture or fresh fodders is important for increasing the vitamin E and essential fatty acid concentrations in muscle tissues.

To stabilize lipid oxidation and colour stability of meat under commercial storage conditions, others proposed that muscle vitamin E concentration in beef (LL) should be at least 3.3 mg/kg meat [41] and ~3.5 mg/kg meat [42], respectively. Previous work on finishing lambs led to vitamin E in meat of about 3.5 mg/kg for diets based on annual pasture and about 6 mg/kg for a perennial pasture diet [36]. With these diets colour stability, as measured by decrease in redness over 72 h of simulated retail display of fresh meat, was about half that measured in the present study (~1 compared to 2.3 a* units). While TBARS was not measured at the start of display in the previous study [36], the TBARS at 72 h of the perennial pasture diet (meat vitamin E ~6 mg/kg) and the annual pasture diet (meat vitamin E ~3.5 mg/kg) are, within experimental error, similar to the TBARS at 1 h in the present study. This compares to the fresh meat TBARS at 72 h in the present study being about thrice the TBARS at 1 h. These observations indicate that meat vitamin E of 3.5 mg/kg is sufficient to prevent lipid oxidation of fresh lamb meat, but meat vitamin E of less than 1 mg/kg is insufficient to prevent lipid oxidation of fresh lamb meat.

As observed in previous studies where lambs were finished on a range of diets, at the time of initial display (1 h) fresh meat had less redness than meat stored for longer periods [43,44]. We propose that with longer storage (ageing) of meat, the muscle (fibres) membrane permeability and integrity weakens. We propose that this allows the oxygen to reach the muscle myoglobin (pigment) faster, resulting initially in a greater redness (cherry red oxymyoglobin) when the meat is cut and displayed for retail assessment. However, in the present study, the lower redness in fresh meat was not maintained from 24 h display onwards.

Irrespective of the three experimental diets, and only marginally affected by whether the meat was yearling or lamb, the decline in redness values between 1 and 72 h display time was 2 units for fresh meat, 6 units for 45 day stored meat, and 8 units for 90 days stored meat. This led to fresh meat displayed for 72 h having comparable colour to meat stored for 45 days after 48 h retail display and meat stored for 90 days after 24 h retail display (Table 3, Figure 1 and Figure 2). This result shows there was a reduction in colour stability (redness) for meat stored for between 45 and 90 days, with the redness being below the recognized threshold of 14.8 for meat to be acceptable for consumers [22] at 72 h for 45 days storage and at 48 h for 90 days storage (Figure 1). It is postulated that the vitamin E concentration of meat was not adequate to delay myoglobin (heme pigment) oxidation in meat stored for 45 and 90 days under semi-frozen condition and then displayed at simulated retail condition for 72 h. A likely explanation is that, at slaughter, all treatments had a vitamin E concentration much below a level of 3–3.5 mg/kg of meat, which is necessary to scavenge free radicals propagated from the polyunsaturated fatty acids (PUFA) or myoglobin (heme pigment) oxidation that enhance the oxidative process in meat post-mortem. A previous study showed that the colour stability of sheep meat as assessed by redness was affected by iron, but not by omega-3 and omega-6 fatty acid concentrations, when the vitamin E concentration of muscle was below this threshold range [45].

The redness over 12 days of retail display of the LL muscle collected at 24 h post-mortem from lambs fed different types of camelina-based lipid supplemented diets, fortified with vitamin E at supranutritional level (360–415 mg/kg diet across treatments) was not affected by dietary treatments [24]; resulting in meat vitamin E concentration ranging from 2.05–4.71 mg/kg across all dietary treatments. In the latter study, muscle samples were stored frozen for 90 days, thawed for 24 h at 4 °C and then packed under oxygen permeable modified atmosphere packaging (sliced 25 mm thickness), using a gas mixture of O_2_:CO_2_ at 80:20% (*v*/*v*), prior to retail display for 12 days at 4 °C. These results indicate that dietary vitamin E fortification protected the meat sample from colour deterioration during retail display, despite the increase in PUFA concentration in the meat caused by chemically protected oilseed and oil supplementation in the diets. In the present study, with low muscle vitamin E concentration (≤ 1 mg/kg meat), the higher PUFA concentration in Merino yearling meat is likely to result in more myoglobin oxidation. This will cause the observed greater decline in meat redness with the Merinos compared to the Composites. All the three diets produced lambs with muscle vitamin E less than 1 mg/kg, which is well below the 3–3.5 mg/kg value needed to prevent lipid oxidation. The minimal effect of diet on colour stability and lipid oxidation, under any storage condition, is likely to be due to the vitamin E in meat being too low to appreciably affect lipid oxidation.

Storage duration and display conditions can influence free radical formation in meat propagated from PUFA, leading to lipid oxidation that, in turn, results in the development of rancid and abnormal flavours in cooked meat. Lipid oxidation evaluated as TBARS values were affected by storage time, retail display time, and animal type, but not by dietary treatments (Table 4). However, a previous study [24] showed TBARS values in meat increased in lambs fed linseed oil or sodium hydroxide treated linseed diets and camelina oil or sodium hydroxide treated camelina seed diets when compared to a Megalac fat supplemented control diet. The PUFA concentration in the LL muscle was also greatest in the linseed diets followed by diets that contained camelina diets and then the control diet supplemented with Megalac. The change in PUFA concentration is most likely the cause of the change in TBARS values across dietary treatments observed in the latter study [24]. However, there was not a close relationship among responses to breed (animal type), storage time, and retail display time for a*-value and TBARS values in the present study. At 1 h retail display, TBARS values were around 0.25 mg MDA/kg meat, irrespective of storage time (fresh or 45 days or 90 days) when compared across animal types (Figure 3) or across all diets (Figure 4). For fresh meat, after 72 h retail display, TBARS values were about 3 times greater than after 1 h retail display (Figure 3; Figure 4) and less than half the critical value of 2 mg MDA/kg meat where off-flavours are developed in meat during cooking [46,47]. In contrast, after 72 h retail display, lamb meat from Composites and yearling meat from Merinos stored for 45 or 90 days had TBARS values about double the critical value for off-flavour development. This is further evidence that the muscle antioxidant status (vitamin E), iron concentration (heme pigment), and fatty acid composition (PUFA) are important factors to maintain colour and sensory attributes of meat, when storing sheep meat under semi-frozen conditions for extended periods.

The increase in ALA in meat observed from animals fed the CAMP diet is considered advantageous for human health because of the consequential increase in the total omega-3 concentration and the decrease in the total omega-6 concentration, whilst maintaining the ratio of n−6/n-3 below 4. As the inclusion of concentrates or grain high in omega-6 increases in sheep diets, the omega-6 FA concentration of meat increases. This, in turn, results in increased PUFA concentration of meat [5,48]. Increasing PUFA concentration of meat through elevated levels of omega-6 concentration would not be beneficial and it has been proposed that the n−6/n−3 ratio in meat should be 4 or less to help maintain a balanced and healthy life [4,49]. The benefits of increasing omega-3 fatty acids has previously been observed with diets containing products derived from camelina seed (camelina oil, camelina meal, camelina seed treated with NaOH, and camelina cake) irrespective of the nutritional level of the diet [11,12,13,24]. However, the omega-3 benefit of supplementing diets with products of camelina seed does not extend appreciably to supplementing diets with camelina hay. This difference is likely associated with the ALA concentration in CAHP (367 mg/100 g of diet) being lower than the ALA concentration in CAMP diet (846 mg/100 g of diet). Whilst there were some smaller effects of diet on the concentration of long chain omega-3 FAs; the concentration of eicosapentaenoic acid (EPA, 20:5n*−3*) plus docosahexaenoic acid (DHA, 22:6n*−3*) was, in all 3 dietary treatments, lower than 30 mg/100 g meat threshold to claim meat as a source of omega-3 [4].

It has previously been shown [45] that PUFA and vitamin E concentrations are negatively associated in the muscle tissue systems of live lambs on a between animal basis, and this relationship did not differ between sheep fed on pasture to sheep fed on pasture supplemented with grain. We suggest, more broadly, that the carcass fatness of an animal could be negatively related to the amount of vitamin E in the muscle. An explanation for such a relationship is that vitamin E in the muscle tissue systems is used to prevent essential fatty acids from oxidation, and high levels of essential fats in the muscle tissues can only be maintained by utilizing vitamin E that is already present in the muscles. Our previous report showed [50] that the carcass fatness of lambs in the current feeding experiment was greater in the diets with camelina hay or camelina meal than in the control diet. Thus, an explanation of the lower muscle vitamin E in the sheep fed the CAHP and CAMP, than in the sheep fed CONP, is that extra muscle vitamin E had been utilized in the live animal to protect the essential fat.

## 5. Conclusions

In this study, lamb and yearling meat produced from sheep supplemented with camelina meal, camelina hay or a grain/hay-based diet had a muscle vitamin E concentration between 0.8 and 1.0 mg/kg. When the meat produced was stored for 45 or 90 days under semi-frozen conditions, this low vitamin E concentration resulted in increased lipid oxidation during 72 h retail display and reduced retail colour stability. These findings reinforce the importance of sheep meat having a vitamin E concentration about 3–3.5 mg/kg muscle to maintain meat colour during retail display, and to avoid off-flavour development of cooked meat due to lipid oxidation, especially for meat stored under semi-frozen conditions for extended periods. This study also confirmed that sheep diets supplemented with camelina meal improved the total omega-3 fatty acid and the ratio of omega-6/omega-3 fatty acid in lamb and yearling meat. This beneficial change in omega-3 fatty acid concentration and omega-6/omega-3 ratio was not observed in lamb and yearling meat produced from sheep supplemented with camelina hay when compared with meat from lambs fed the control diet.

## Figures and Tables

**Table 1:** Dietary ingredients, nutritive characteristics, vitamin E, α-linolenic acid, and linoleic acid concentrations of pelleted diets fed to Maternal Composite lambs and Merino yearlings.

Dietary Ingredients Used (%)	Camelina Hay (CAHP) ^1^	Camelina Meal (CAMP) ^2^	Control Pellet (CONP) ^3^
Camelina meal	0	8	0
Lupins	30	22	30
Barley grain	10	20	20
Oat grain	15	5	5
Oaten hay	0	45	45
Camelina–Oaten–Barley hay (33:33:33 *w*/*w*/*w*)	45	0	0
Nutritive characteristics of diet			
Dry matter, %	88.85	88.90	89.35
Crude protein, % DM	15.20	14.93	14.80
Metabolisable energy, MJ/ kg DM	10.83	11.23	10.80
Crude fat, % DM	2.91	3.65	2.11
Acid detergent fibre, % DM	19.93	17.78	19.03
Neutral detergent fibre, % DM	34.03	31.18	34.23
Lignin, % DM	4.20	4.13	4.30
Phosphorous, % DM	0.42	0.52	0.47
Potassium, % DM	1.42	1.52	1.47
Sulphur, % DM	0.21	0.26	0.25
Vitamin E concentration, mg/kg DM	4.1	4.2	4.9
Linolenic acid (n − 3) concentration, mg/100g DM	367	846	228
Linoleic acid (n − 6) concentration, mg/100 g DM	1433	1391	1357

^1^ CAHP = Pelleted diet containing camelina hay; ^2^ CAMP = Pelleted diet containing camelina meal; ^3^ CONP = control diet containing cereal hay and grains; DM = dry matter.

**Table 2:** Effect of diet and animal type on vitamin E concentration, meat tenderness and individual and major group fatty acids in muscle *longissimus lumborum*. Fatty acid concentrations reported in parentheses are reported in mg/100 g meat from back-transformed log_10_ mean fatty acid values.

	Effect of Diet				Effect of Animal Type			*p*-Value		
	CAHP ^1^	CAMP ^2^	CONP ^3^	Sed ^4^	Composite	Merino	Sed ^4^	Diet	Breed	Interaction of Diet × Breed
Vitamin E (mg/kg meat)	0.81	0.86	0.99	0.029–0.031	0.76	1.04	0.024	**4.4 × 10^−5^**	**1.1 × 10^−8^**	0.25
Meat tenderness (N)	34.9	36.3	33.1	2.55–2.72	33.3	35.9	2.11	0.47	0.24	0.73
**Fatty acid content** (log_10_ transformed; back-transformed means in parentheses are mg/100 g meat)										
C10:0	0.68 (4.7)	0.68 (4.8)	0.69 (4.9)	0.025–0.026	0.56 (3.6)	0.81 (6.5)	0.020	0.77	**4.6 × 10^−9^**	0.70
C12:0	0.53 (3.4)	0.56 (3.6)	0.57 (3.7)	0.031–0.034	0.58 (3.8)	0.52 (3.3)	0.026	0.47	**0.036**	0.74
C14:0	1.93 (86)	1.97 (92)	1.94 (87)	0.023–0.024	1.88 (76)	2.01 (103)	0.023	0.41	**4.4 × 10^−6^**	0.42
C14:1	0.41 (2.6)	0.41 (2.6)	0.43 (2.7)	0.039–0.042	0.32 (2.1)	0.52 (3.3)	0.033	0.80	**2.9 × 10^−5^**	0.76
C15:0	0.98 (9.5)	0.99 (9.8)	0.99 (9.8)	0.026–0.028	0.88 (7.6)	1.09 (12.4)	0.022	0.90	**1.4 × 10^−7^**	0.75
C15:1	0.21 (1.6)	0.12 (1.3)	0.20 (1.6)	0.039–0.042	0.26 (1.8)	0.09 (1.2)	0.032	0.091	**0.00012**	0.25
C16:0	2.98 (960)	3.01 (1020)	2.99 (970)	0.014–0.015	2.88 (760)	3.10 (1270)	0.012	0.23	**2.5 × 10^−11^**	0.13
C16:1	1.79 (61)	1.79 (61)	1.80 (63)	0.026–0.027	1.65 (44)	1.94 (86)	0.021	0.83	**1.8 × 10^−9^**	0.70
C17:0	1.50 (31)	1.50 (32)	1.50 (32)	0.017–0.018	1.37 (23)	1.63 (42)	0.014	0.90	**2.6 × 10^−11^**	0.65
C18:0	2.79 (617)	2.80 (632)	2.79 (613)	0.015–0.016	2.70 (503)	2.88 (764)	0.013	0.69	**9.3 × 10^−10^**	0.34
C18:2n−6 *cis*	2.14 (136)	2.33 (216)	2.00 (99)	0.040–0.042	1.94 (86)	2.34 (220)	0.033	**2.9 × 10^−5^**	**5.7 × 10^−9^**	0.23
C18:1n-9 *cis*	3.20 (1600)	3.20 (1600)	3.21 (1610)	0.011–0.012	3.09 (1230)	3.32 (2090)	0.009	0.90	**6.0 × 10^−13^**	0.019
C20:0	0.56 (3.6)	0.67 (4.7)	0.53 (3.4)	0.018–0.019	0.52 (3.3)	0.64 (4.4)	0.015	**4.1 × 10^−6^**	**1.4 × 10^−6^**	0.050
C18:3n−6	−0.24 (0.6)	−0.57 (0.3)	−0.13 (0.7)	0.051–0.055	−0.38 (0.4)	−0.20 (0.6)	0.042	**1.8 × 10^−6^**	**0.00087**	0.12
C18:3n−3 (ALA ^5^)	1.43 (27)	1.74 (55)	1.40 (25)	0.020–0.022	1.38 (24)	1.64 (44)	0.017	**2.2 × 10^−10^**	**4.6 × 10^−10^**	0.37
C20:4n−6 (AA ^6^)	1.51 (32)	1.45 (28)	1.54 (35)	0.009–0.010	1.54 (35)	1.47 (29)	0.008	**2.9 × 10^−7^**	**1.5 × 10^−7^**	0.46
C20:5n−3 (EPA ^7^)	1.12 (13.1)	1.56 (14.3)	1.14 (13.9)	0.010–0.011	1.14 (13.7)	1.14 (13.8)	0.008	**0.0075**	0.87	0.17
C22:5n−3 (DPA ^8^)	1.24 (17.3)	1.26 (18.1)	1.27 (18.6)	0.009	1.25 (17.9)	1.26 (18.3)	0.007	**0.011**	0.21	0.43
C22:6n−3 (DHA ^9^)	0.65 (4.5)	0.66 (4.5)	0.71 (5.2)	0.016–0.017	0.62 (4.1)	0.74 (5.5)	0.013	**0.0018**	**2.8 × 10^−7^**	0.062
EPA + DHA	1.25 (18)	1.28 (19)	1.28 (19)	0.010–0.011	1.25 (18)	1.28 (19)	0.008	**0.0072**	**0.0016**	0.094
EPA + DPA + DHA	1.54 (35)	1.57 (37)	1.58 (38)	0.009	1.55 (36)	1.57 (38)	0.007	**0.0052**	**0.011**	0.17
Total n-6 (Omega-6)	2.22 (166)	2.21 (161)	2.23 (171)	0.009–0.010	2.19 (156)	2.25 (177)	0.008	**0.038**	**4.9 × 10^−6^**	0.033
Total n−3 (Omega-3)	1.80 (62)	1.97 (94)	1.81 (64)	0.013–0.014	1.79 (62)	1.92 (83)	0.011	**4.1 × 10^−9^**	**1.5 × 10^−8^**	0.24
n−6/n−3	0.43 (2.7)	0.24 (1.7)	0.43 (2.7)	0.011	0.41 (2.5)	0.34 (2.2)	0.009	**5.5 × 10^−11^**	**2.3 × 10^−6^**	0.88
∑ SUFA ^10^	3.23 (1720)	3.25 (1800)	3.24 (1720)	0.013–0.14	3.14 (1380)	3.34 (2200)	0.011	0.32	**3.3 × 10^−11^**	0.16
∑ MUFA ^11^	3.26 (1800)	3.28 (1880)	3.25 (1780)	0.012–0.013	3.14 (1370)	3.38 (2420)	0.010	0.15	**5.2 × 10^−13^**	0.028
∑ PUFA ^12^	2.36 (228)	2.41 (255)	2.37 (235)	0.010	2.34 (219)	2.42 (261)	0.008	**0.00089**	**1.3 × 10^−7^**	0.023
Total fat	3.57 (3750)	3.60 (3940)	3.57 (3750)	0.012–0.013	3.47 (2970)	3.69 (4880)	0.010	0.18	**3.2 × 10^−12^**	0.077

^1^ CAHP = Pelleted diet containing camelina hay; ^2^ CAMP = Pelleted diet containing camelina meal; ^3^ CONP = control diet containing cereal hay and grains. ^4^ Sed = standard error of difference. ^5^ ALA = alpha-linolenic acid (n−3), ^6^ AA = arachidonic acid (n−6), ^7^ EPA = eicosapentaenoic acid (n−3), ^8^ DPA = docosapentaenoic acid (n−3), ^9^ DHA = docosahexaenoic acid (n−3), ^10^ ∑ SUFA (saturated fatty acid) = C10:0 + C12:0 + C14:0 + C15:0 + C16:0 + C17:0 + C18:0 + C20:0, ^11^ ∑ MUFA (monounsaturated fatty acid) = C14:1 + C15.1 + C16:1 + C18:1n-9 and ^12^ ∑ PUFA (polyunsaturated fatty acid) = C18:2n−6 + C18:3n−3 + C18:3n−6 + C20:4n−6 + C20:5n−3 + C22:5n−3 + C22:6n−3. *p*-values less than 0.05 are in bold.

**Table 3:** Repeated measures analysis of variance for the redness (a*-value) of muscle *longissimus lumborum* displayed for 72 h under simulated refrigeration conditions. Degrees of freedom are corrected by the Greenhouse–Geisser epsilon in the between display times stratum (ε = 0.6204; *p* = 0.00002 for testing difference from 1).

Terms	Degrees of freedom	Mean Square	F-Value	*p*-Value
Between pens				
Animal type (AT)	1	19.054	9.36	**0.0085**
Diet	2	0.039	0.02	0.98
Diet. AT	2	0.416	0.20	0.82
Error	14	2.034		
Between storage lengths within pens combinations				
Storage length (ST)	2	82.66	145.57	**1.6 × 10^−16^**
AT. ST	2	0.159	0.28	0.76
Diet. ST	4	0.781	1.37	0.27
Diet. AT. ST	4	0.914	1.61	0.20
Error	28			
Between display times within storage lengths within pens				
Display time (DT)	1.86	361.89	2616.4	**2.9 × 10^−71^**
ST. DT	3.72	35.324	255.39	**4.2 × 10^−43^**
AT. DT	1.86	5.446	39.37	**4.6 × 10^−12^**
AT. ST. DT	3.72	0.103	0.75	0.56
Diet. DT	3.72	0.085	0.61	0.64
Diet. ST. DT	7.44	0.055	0.40	0.91
Diet. AT. DT	3.72	0.600	4.34	**0.0039**
Diet. AT. ST. DT	7.44	0.060	0.44	0.89
Error	78.17			

*p-*values less than 0.05 are in bold.

**Table 4:** Analysis of variance for the square root of TBARS of muscle *longissimus lumborum* subjected to different storage times (ST) and different display times (DT).

Terms	Degrees of Freedom	Mean Square	F-Value	*p*-Value
Between pens				
Animal type (AT)	1	0.002	0.00	0.96
Diet	2	0.011	0.17	0.85
Diet.AT	2	0.009	0.13	0.88
Error	14	0.067		
Between storage lengths within pens combinations				
Storage times (ST)	2	3.692	556.38	**2.9 × 10^−23^**
AT.ST	2	0.165	24.80	**6.3 ×10^-7^**
Diet.ST	4	0.011	1.58	0.21
Diet.AT.ST	4	0.009	1.32	0.29
Error	26	0.007		
Between display times within storage lengths within pens				
Display time (DT)	1	36.18	1889.7	**1.5 × 10^-36^**
ST.DT	2	3.69	192.9	**6.8 × 10^-22^**
AT.DT	1	0.010	0.54	0.47
AT.ST.DT	2	0.064	3.32	**0.046**
Diet.DT	2	0.028	1.47	0.24
Diet.ST.DT	4	0.002	0.12	0.97
Diet.AT.DT	2	0.020	1.05	0.36
Diet.AT.ST.DT	4	0.018	0.93	0.46
Error	42			

*p-*values less than 0.05 are in bold.

## Data Availability

The data presented in this study are available on request from the corresponding author. The data are not publicly available due to the data being prepared for further publication.
